# Development and Validation of a Questionnaire to Measure Medication Adherence to Direct-Acting Agents in Patients with Hepatitis C

**DOI:** 10.3390/pharmaceutics13101683

**Published:** 2021-10-14

**Authors:** Adina Turcu-Stiolica, Irina Paula Doica, Bogdan Silviu Ungureanu, Ion Rogoveanu, Dan Nicolae Florescu, Mihaela-Simona Subtirelu, Dan Ionut Gheonea

**Affiliations:** 1 Pharmacoeconomics Department, University of Medicine and Pharmacy of Craiova, 200349 Craiova, Romania; adina.turcu@umfcv.ro (A.T.-S.); mihaela.subtirelu@umfcv.ro (M.-S.S.); 2 Gastroenterology Department, University of Medicine and Pharmacy of Craiova, 200349 Craiova, Romania; ion.rogoveanu@umfcv.ro (I.R.); dan.florescu@umfcv.ro (D.N.F.); dan.gheonea@umfcv.ro (D.I.G.)

**Keywords:** adherence in hepatitis C, direct-acting antivirals, development and validation

## Abstract

This study aims to develop a new self-report tool (HCV-AD) measuring adherence factors, intentional or unintentional, during Hepatitis C Virus (HCV) treatment with direct-acting antivirals (DAA) aiming to achieve high efficacy, otherwise resulting in drug resistance and treatment failure. Two phases were conducted: in the first phase, items were generated based on an extensive literature review, and, in the second phase, a prospective cohort study was conducted using HCV patients from Gastroenterology Department from University County Hospital of Craiova, Romania (n = 222), to evaluate the validity and reliability of the questionnaire. A number of 19 items were generated following a systematic review and through expert opinion. The internal consistency reliability was evaluated using Cronbach’s alpha. The construct validity was assessed using correlations with two other instruments: visual analog scale (VAS) and medication possession ratio (MPR). The final questionnaire (HCV-AD10) was derived through exploratory factor analysis, with 82% of total variance explained. This instrument appeared as a reliable and valid measure for medication adherence, with Cronbach’s alpha (0.867) and significant high positive correlations between adherence scores calculated with HCV-AD10 and VAS (ρ = 0.61, *p* < 0.001) or with HCV-AD10 and MPR (ρ = 0.75, *p* < 0.001). This research would make a worthwhile contribution to HCV management.

## 1. Introduction

Direct-acting antivirals (DAA) have resulted in path breaking development of hepatitis C virus (HCV) management [1]. HCV was discovered just before the 1990s [2] and similar to other gastrointestinal diseases which might be preventable by other techniques [3], determining its serology and replication became essential for everyday practice, especially for patients receiving blood transfusions. The World Health Organization (WHO) target of HCV elimination in European Countries [4] seems rather feasible, as many countries have focused their attention on micro-screening [5] and extended the panel of DAA to cover all possible downsizing, such as phenotypic variation or medication adherence [6]. While they are well tolerated, with a course of therapy of 12 weeks depending on the fibrosis stage, their action is still related to medication adherence [7].

HCV management with DAA lacks in measurements studies, with only a few evidence-based methods included so far. Medication adherence is known to be influenced by negative perceptions about medication, poor knowledge, high costs or fear of possible side effects [8]. While the era of interferon regimens focused on these issues, the new DAA may face a challenge in specific situations due to the psychosocial or socio-economic burden that HCV patients may encounter [9]. In addition, promoting new medication for patients who underwent interferon therapy and had severe side effects might make them reluctant at first.

Despite the high success of therapy, DAA agents still require valid tools to assess their adherence in clinical practice. The threshold of 80% has been used to characterize a good adherence since 1976 [10], but a new threshold must be defined for various therapeutic areas [11], being clearly questioned as a general standard [12]. As there is no documented standard of DAA adherence to obtain cure rates [13], a new valid and clinically useful threshold could be established for DAA non-adherence from which a patient no longer could have good outcomes in HCV treatment. Studies so far have focused on pill counts (PC) [14], the use of Visual Analog Scale (VAS) [15] and mostly pharmacy records [16,17], which help to construct a general idea in specific situations over medication adherence. The number of wireless pillbox openings or video-recorded ingestions was used for DAA adherence monitoring [18]. However, other factors should be considered too, such as previous treatment experience, which may generate medication administration fear, as well as multi-tablet therapy by adding more medication to their previous treated comorbidities [19]. Moreover, the concept of medication possession ratio (MPR) does not cover the period when patients might discontinue their therapy earlier than described [20].

The aim of our study is to develop a reliable and valid adherence self-report tool, easy to administer on a large scale, while attending to the main limitations of currently used tools in DAA adherence of HCV patients.

## 2. Materials and Methods

Within a prospective study on HCV patients, we developed a new tool for assessing self-reported adherence, by starting with item generation and content validity, followed by a preliminary questionnaire development, and concludes with a rigorous scientific evaluation, succeeding by implementing the following steps: pilot testing, data collection (sampling and survey administration), item reduction analysis and evaluation [21,22]. Data on socio-demographic patient characteristics, education, employment, and income, were obtained at baseline. Medication-related beliefs, evaluated as factors potentially associated with non-adherence, were collected at 30 days post the DAA treatment. The study was approved by the Ethics Committee of the University of Medicine and Pharmacy of Craiova, Romania (no. 87/12.02.2020).

### 2.1. Generation of the HCV-AD Items

The items of the questionnaire used for DAA adherence assessment in HCV patients (HCV-Adherence, HCV-AD) were proposed based on a prior literature review of self-reported adherence, followed by a careful analysis of the content validity by two experts (B.S.U. and A.T-S.). We included many items to better capture all the factors influencing medication adherence. The questions were arranged and reworded in order to eliminate ambiguity, and technical jargon. A pilot testing of the proposed instrument was conducted in the next step in the target population (a sample of 30 HCV patients).

### 2.2. HCV-AD Evaluation

The Kaiser–Meyer–Olkin (KMO) criterion was applied for testing the appropriate sample size. The determinant and Bartlett’s test of sphericity were used to establish the stability of the factors. Reliability and validity were evaluated. Factors were extracted and optimized through Exploratory Factor Analysis (EFA). Kaiser’s criterion and Cattell’s scree plot were used, a factor with more than one eigenvalue was retained after the elbow indicated the cut-off point for factor extraction. Internal consistency was assessed through Cronbach’s alpha (α) coefficient. Construct validity was demonstrated by correlation matrices of associations between HCV-AD score and two other instruments measuring adherence: VAS and MPR. The VAS recorded the respondent’s self-related adherence where the endpoints are labelled “100% adherent” and “0% adherent”, being a quantitative measure of medication adherence as judged by the individual respondents. We used individual patient data reported by community pharmacies at the Health Insurance House, following the legal requirements on personal data protection, to calculate the medication adherence with MPR formula:

$$\mathrm{Adherence}=\frac{\sum^{\;}tablets\;dispersed}{\sum^{\;}tablets\;prescribed}$$

### 2.3. Sample

Patients with detectable RNA-HCV at baseline, regardless of the value, were considered eligible. We did not exclude patients which followed previous treatment with interferon, which either did not respond to treatment or were or stopped the treatment due to side effects. We excluded individuals with HIV- coinfection, or recently diagnosed with malignancies.

**Figure 1:**
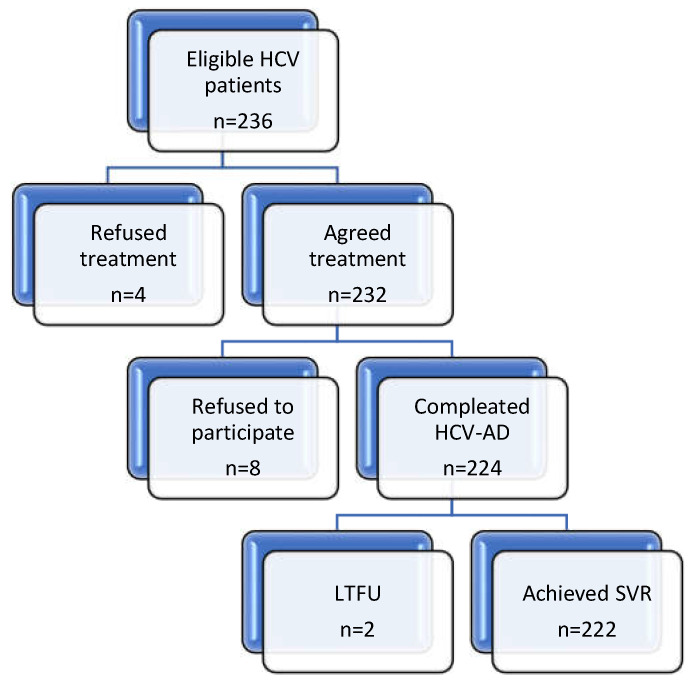
Study design and participant flow. HCV, hepatitis C virus; HCV-AD, the medication adherence questionnaire; LTFU, lost to follow-up; SVR, sustained virologic response.

A total of 236 were enrolled across the University County Hospital of Craiova, Romania, from March 2020 to April 2021, as in Figure 1. Of the eligible HCV patients, 4 refused DAA treatment, 8 refused to participate, and 2 patients were lost to follow-up.

### 2.4. Statistical Analysis

Continuous variables were expressed as means (standard deviation), and categorical variables as frequencies (percentage). Data management and analysis, and exploratory factor analysis (EFA) were done using GraphPad Prism 9.1.0 (GraphPad Software, San Diego, CA, USA). Prior to the extraction of the factors, Kaiser–Meyer-Olkin (KMO) Measure of Sampling Adequacy and Bartlett’s Test of Sphericity were determined. A KMO index of 0.5 and significant Bartlett’s test of sphericity (*p* < 0.01) were considered suitable for factor analysis [23]. Parallel analysis was conducted using EFA.dimensions package in R software (R foundation, Vienna, Austria). Once the final model was determined, the internal consistency was tested using Cronbach’s α and McDonald’s Omega [24] using the MBESS package in R software [25,26]. The threshold of >0.8 was considered as having high internal consistency (>0.7 is a good consistency, <0.7 is considerably questionable). Convergent validation of the questionnaire was examined using Spearman correlation coefficients. A *p*-value less than 0.05 was considered statistically significant.

## 3. Results

The paper-based questionnaire with 20 initial items was administered for pre-testing in the pilot step to 30 patients with HCV to assess item clarity and to estimate the reliability. Patients confirmed items as readable and accurate in reflecting the factors influencing medication adherence. One item was dropped for lack of importance. Items were positively stated with three exceptions (Q11, Q12, Q18); the negatively stated items were reverse coded.

The range for completing the survey was between 5 and 10 min.

A sample of 222 HCV patients completed all survey items, including 25% were male. More details about the sample characteristics can be seen in Table 1.

### 3.1. Exploratory Factor Analysis

The initial questionnaire proposed 19 items (Table 2) with five response options (also called the five-point Likert scale, ranked from “Strongly disagree” to “Strongly agree” (Q5, Q6, Q17) or from “Never” to “Very often” (Q1–Q4, Q7–Q16). The last question Q19 has “yes/no” answers. Every item has the score from 5 to 1.

An EFA was used to reduce the items to a more parsimonious set for measuring DAA adherence for HCV patients. Two questions were deleted (Q2 and Q7) because their variance was null. The sample size measure through KMO (0.834) and Bartlett’s test of sphericity (*p* < 0.001) confirmed that the item scores were suitable for factor analysis.

**Figure 2:**
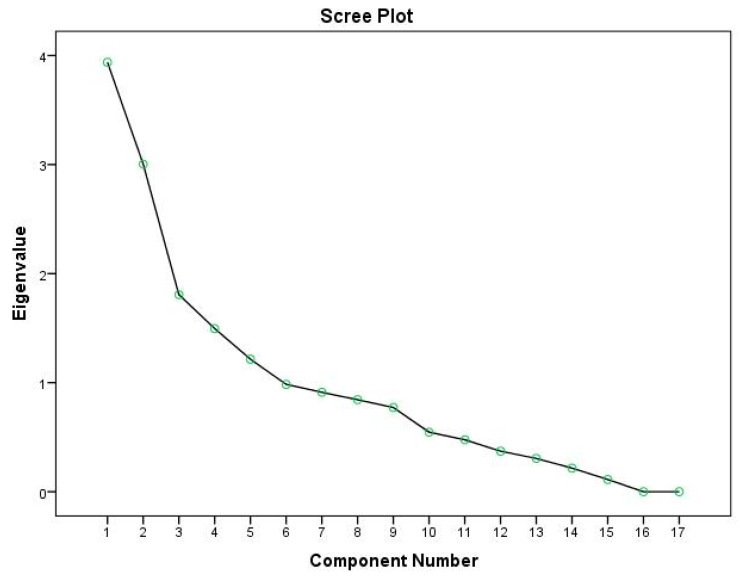
Scree plot of Exploratory Factor Analysis.

In the first round of EFA, the responses on the 17 items of HCV-AD were intercorrelated and rotated to an orthogonal solution. The eigenvalues and the scree plot (Figure 2) were examined, providing a number of five factors represented by the data, which were verified also with parallel analysis (Table 3).

Next, the items included in the extracted five factors were reviewed. Factors with less than three items were removed to ensure that each factor was well measured. Reliability was assessed for every factor: if Cronbach’s α was greater than 0.7, all the items remained into that factor. Factor 1 (Q1, Q3, Q4, Q9) had α = 0.888 and we included them in the final questionnaire. Factor 2 (Q5, Q16, Q17, Q18) had α = 0.681 and after Q16 and Q18 were deleted, α = 0.732. Factor 3 (Q6, Q12, Q14) had α = 0.720 and after Q12 was deleted, α = 0.864. Factor 4 (Q8, Q11, Q13) had α = 0.786 and after Q11 was deleted, α = 0.831. Factor 5 contained only two items (Q10, Q15). The other items were not included in factors.

The analysis yielded three factors that accounted for 70% of the total variance with Factor 1 explaining 31.55% of the variance whereas Factor 2 explaining 24.8%, as seen in Table 4.

As in Table 5, the three subscales that emerged from the EFA represent the areas of medication adherence: unintended factors (forgetting to take or to have the drugs when they had to take them), fear of the drugs, the intended factors (if the patients feel sick or better, they do not take the drugs).

The adherence score after applying the 10-items HCV-AD will be calculated as the sum of all scored items minus 10 and divided by 0.40:

$$\mathrm{HCV}-\mathrm{AD}10\;\mathrm{score}=\frac{\sum_{i=1}^{10}Qi-10}{0.40}$$

### 3.2. Internal Consistency

In the total sample, the Cronbach’s alpha coefficient for the total HCV-AD10 was 0.867 and item-total correlations ranged from 0.17 to 0.79. None of the 10 final items could be deleted without a decrease in Cronbach’s alpha. The McDonald’s omega value (ω = 0.877) confirmed that the HCV-AD10 is high.

### 3.3. Construct Validity

To investigate whether HCV-AD10 captures medication adherence, we examined the correlations (Spearman’s ρ) between the obtained scores and VAS and MRP scores. Descriptive statistics of adherence scores are presented in Table 6.

HCV-AD19, the initial questionnaire with 19 items; HCV-AD10, the final questionnaire with 10 items; VAS, visual analog scale; MPR, medication possession ratio.

**Figure 3:**
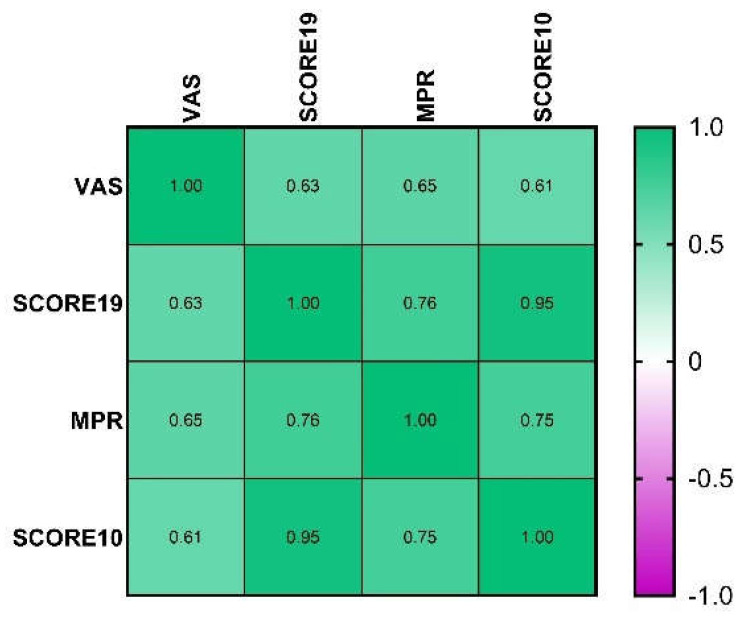
Heatmap containing Spearman coefficients with values from −1 to 1, as the colours from the right show, larger values were represented by dark green and smaller values by dark mauve. SCORE10, the adherence score calculated with HCV-AD10; SCORE19, the adherence score calculated with HCV-AD19.

Spearman’s correlation analysis demonstrated significant positive correlations between adherence scores calculated with HCV-AD10 (SCORE10) and VAS (ρ = 0.61, *p* < 0.001) or with HCV-AD10 and MPR (ρ = 0.75, *p* < 0.001), as shown in Figure 3.

## 4. Discussion

Our HCV-AD10 questionnaire has good construct validity, supporting its adequacy for jeopardizing the effectiveness of DAA therapy in patients with HCV. To the best of our knowledge, this is the first questionnaire trying to understand the factors expression which facilitates or sabotages medication adherence in patients with HCV when DAA are used. The other tools used to assess the DAA adherence did not reveal why the patients do not adhere to the prescribed treatment. Serper et al., assessed the DAA adherence for HCV considering non-adherence being the absence of lab results after DAA therapy completion [27]. However, taking medication is a complex behaviour and pharmacy refill analysis is not practical due to the short duration of the treatment.

Butt et al., demonstrated that the efficacy of sofosbuvir-based treatment regimens was not affected when patients were nonadherent [28]. Even though this new line of therapy used for HCV has overcome the boundaries of interferon regimens, the path for HCV eradication might still require substantial tools to achieve high medication adherence. HCV-AD is a practical adherence method, easy to apply and might determine potential patients who may be reluctant to therapy.

We proposed a new instrument that might aid in identifying a specific group of patients, which might require extensive monitoring over their treatment time. DAA offer several advantages when administered. Currently, there are many available options that may cover all genotypes and might successfully lead to HCV elimination. If used, these pan-genotypic regimens might be very helpful for HCV eradication. Moreover, the fibrosis stage could also be considered an important factor for medication adherence as it may reduce pills administration from 12 to 8 weeks. The patients included in this study proved to have high medication adherence, which may be related to the single genotype and the fact that most patients were in a lower fibrosis stage, which means only 8 weeks of therapy. However, all these reasons might not be the actual main factors for medication adherence when using DAA for HCV treatment.

Most of the HCV patients could be reluctant at first, as they are more familiar with past interferon therapy which had significant side effects and also a low response rate. Perhaps the fear of the unknown for the new DAA might represent the most significant potential drawback when considering following medication, even if it is for a small period of time. The side effects of Interferon, as well as the use of new pills, might instate anxiety or fear of potential adverse reaction. Thus, identifying these types of risk patients could help prevent medication non-adherence. On the other hand, most of the patients with HCV present with other diseases which require extensive medication, which could require an extensive panel of medication per day. Adding more pills, even for a small amount of time, could confuse the elderly and make them even more unwilling to follow the treatment. Moreover, some DAAs may interact with patient’s chronic medication, which might confuse them as they may need to replace some drugs during HCV eradication treatment. Hence, applying a targeted questionnaire could prevent some of these issues by focusing on specific aspects to encourage the patient to follow the DAA treatment.

We propose this new instrument to assess medication adherence on DAA, as it may provide a new perspective to include and understand even the most reluctant patients who for some reason might not follow the medication as indicated. Providing HCV-AD before treatment initiation could measure the patient’s understanding of the DAA’s risks and benefits and could lead to further explanation by the physician to overcome possible flaws. Our tool is a self-reported method which might be safely used to cover a rather short period of treatment either 8 or 12 months. It is inexpensive and according to our results serves as a reliable method to assess HCV patient’s adherence. It is noteworthy that HCV-AD may not only be used only by prescribing physicians, but also by pharmacists, since studies have shown their potential role in medication adherence [17]. Involving a multidisciplinary team could raise patients trust and understanding of DAA for HCV management.

Our study also has several limitations. First, developing an HCV adherence tool should contain a diversity of patient’s characteristics such as different genotypes, more types of DAA and a larger number of patients. Our cohort of HCV patients is rather inhomogeneous, with most of them being elderly and retired from work. This may be related to the fact that most of these patients did not address the previous national HCV therapy programs and were either found by micro screening programs or newly diagnosed on baseline analysis. Even though the specific DAA used in this study are still on the market, new DAA have been included and will be more frequently used since they will cover up all genotypes and be more safely used in specific patients such as the ones with end stage kidney disease, HIV associated disease or even children. On the other hand, all included patients were treated in a single medical centre. The 5-Likert rating scale, we chose, allows answers that are not undermined by forced completion and, also, the capacity to generate sufficient variance among the intended respondents. No tests of structural validity were performed, as the questions were not hypothesized to reflect latent dimensions of adherence. The DAA treatment is for a short period of time, with very promising outcomes, common reliability, and validity tests (e.g., Cronbach’s αfactor analysis) being very well applicable to stable behaviour [29].

Because all patients had SVR of 100% we could not establish a threshold for DAA adherence. However, we are convinced that by having this instrument, future studies must be conducted to assess how small the adherence level can be, as not to achieve high virological response and sustained virological response.

This instrument might be a helpful tool in the WHO’s objective of HCV eradication as it may cover some patients who may be non-adherent to therapy, exploring its predictors and finding strategies to promote DAA adherence.

## 5. Conclusions

Our developed HCV-AD10 is a validated and reliable instrument for evaluating adherence among HCV patients using the DAAs in Romania. It is simple to use and suitable to assess the patient-specific, illness-specific, or medication-related factors which are barriers to HCV eradication.

## Figures and Tables

**Table 1:** Baseline characteristics of patients.

Characteristics	Patients (n = 222)
Age in years, mean (SD)	60.8 (12.1)
Gender–Male, n (%)	56 (25.2%)
Education	
Elementary school High school Faculty	114 (51.4%) 88 (39.6%) 20 (9.0%)
Marital status	
Married Single, never married Widowed Divorced	162 (73%) 12 (5.4%) 40 (18%) 8 (3.6%)
Environmental	
Urban Rural	92 (41.4%) 130 (58.6%)
Employment status	
Employed Unemployed Social help Retired	64 (28.8%) 14 (6.3%) 6 (2.7%) 138 (62.2%)
Income	
<2000 RON 2000–4000 RON 4000–6000 RON >6000 RON	186 (94.6%) 34 (15.3%) 2 (0.90%) 0
Smoking	
Former smoker Smoker Never smoker	44 (19.8%) 24 (10.8%) 154 (69.4%)
HCV genotype	
3b	222 (100%)
HCV duration in years, mean (SD)	6.59 (6.9)
Treatment regimen	
Ledipasvir/sofosbuvir Dasabuvir/ombitasvir/paritaprevir/ritonavir	152 (68.5%) 70 (31.5%)
Treatment duration, n (%)	
8 weeks 12 weeks	112 (50.5%) 110 (49.5%)
SVR, sustained virologic response	222 (100%)
Fibrosis stage, n (%)	
F0 F0–F1 F1 F1–F2 F2 F2–F3 F3 F3–F4 F4	28 (12.6%) 10 (4.50%) 18 (8.1%) 36 (16.2%) 26 (11.7%) 2 (0.9%) 18 (8.1%) 10 (4.5%) 74 (33.3%)

**Table 2:** The HCV-AD19.

Questions of Initial Questionnaire HCV-AD	Reasons
**1. In the last week, I forgot to take the prescribed medicines.**	
2. In the last week, I was too busy to take the prescribed medicines.	Deleted (variance = 0)
**3. The medicines caused side effects and I did not take them as doctor prescribed them.**	
**4. In the last week, I did not have my medicines with me when I had to take them.**	
**5. I am not sure the medicine will make me feel better.**	
**6. I take too many medicines.**	
7. In the last week, I have modified the dose of my medicines.	Deleted (variance = 0)
**8. When I feel better, I do not take my medicines for a while.**	
**9. In the last week, I skipped a dose of my medicines.**	
10. I avoid taking my medicines because I do not know how they work.	
**11. I take my medicines every day**.	
12. I am incredibly careful not to forget to take my medicines.	
**13. I do not take my medicines when I feel too sick.**	
**14. I am afraid not to become addicted on my medicines.**	
15. I forget to take my prescription when I was scheduled for it.	
16. I do not like to take medications. If I can work without taking them, then I do not take them.	
**17. I do not expect miracles after starting my treatment.**	
18. I strictly respect what the doctor tells me.	
19. I know how to take my medicines.	

Final items of HCV-AD were bolded.

**Table 3:** The total variance explained with Eigenvalues using EFA.

Factor	Initial Eigenvalues			Rotation Sums of Squared Loadings			Means from Parallel Analysis
	Total	% of Variance	Cumulative %	Total	% of Variance	Cumulative %	
1	3.94	23.17	23.17	3.17	18.64	18.64	1.5218
2	3.00	17.67	40.73	2.3	13.55	32.19	1.4069
3	1.81	10.63	51.46	2.17	12.78	44.97	1.3319
4	1.5	8.8	60.26	2.09	12.3	57.27	1.2555
5	1.22	7.15	67.41	0.72	10.14	67.41	1.1949
6	0.98	5.79	73.2				1.1346
7	0.91	5.36	78.56				1.0779
8	0.84	4.96	83.53				1.0261
9	0.77	4.54	88.07				0.9757
10	0.55	3.21	91.28				0.9253
11	0.48	2.8	94.09				0.8789
12	0.37	2.19	96.27				0.8345
13	0.31	1.79	98.07				0.7889
14	0.22	1.27	99.34				0.744
15	0.11	0.66	100				0.6918
16	4.8*10^−33^	2.8*10^−32^	100				0.6418
17	−9.6*10^−17^	−5.7*10^−16^	100				0.5692

**Table 4:** The total variance explained with Eigen values for the HCV-AD10.

Factor	Initial Eigenvalues			Rotation Sums of Squared Loadings		
	Total	% of Variance	Cumulative %	Total	% of Variance	Cumulative %
1	3.28	32.77	32.77	3.16	31.55	31.55
2	2.47	24.73	57.51	2.48	24.8	56.35
3	1.29	12.95	70.45	1.41	14.10	70.45
4	0.94	9.44	79.89			
5	0.86	8.55	88.44			
6	0.56	5.63	94.07			
7	0.46	4.59	98.66			
8	0.13	1.34	100			
9	1.4*10^−33^	1.4*10^−32^	100			
10	−7.8*10^−17^	−7.8*10^−16^	100			

**Table 5:** Factor loadings for the survey items after Exploratory Factor Analysis.

Survey Item	Subscale (Factors)		
	1	2	3
1. In the last week, I forgot to take the prescribed medicines.	0.887		
3. The medicines caused side effects and I did not take them as the doctor prescribed them.	0.887		
4. In the last week, I did not have my medicines with me when I had to take them.	0.883		
9. In the last week, I skipped a dose of my medicines.	0.883		
5. I am not sure the medicine will make me feel better.		0.773	
17. I do not expect miracles after starting my treatment.		0.712	
6. I take too many medicines.		0.853	
14. I am afraid not to become addicted to my medicines.		0.787	
8. When I feel better, I do not take my medicines for a while.			0.846
13. I do not take my medicines when I feel too sick.			0.813

**Table 6:** Adherence scores calculated with the used scales.

Adherence Scales	Adherence Scores		
	Mean	SD	Range
HCV-AD19	93.99	5.63	80.26–100
HCV-AD10	91.53	8.42	72.5–100
VAS	9.78	0.49	8–10
MPR	96.7	4.93	87.5–100

## Data Availability

The datasets used and/or analyzed during the current study are available from the corresponding authors on reasonable request.
